# Mutagenicity and Pollutant Emission Factors of Solid-Fuel Cookstoves: Comparison with Other Combustion Sources

**DOI:** 10.1289/ehp.1509852

**Published:** 2016-02-19

**Authors:** Esra Mutlu, Sarah H. Warren, Seth M. Ebersviller, Ingeborg M. Kooter, Judith E. Schmid, Janice A. Dye, William P. Linak, M. Ian Gilmour, James J. Jetter, Mark Higuchi, David M. DeMarini

**Affiliations:** 1 National Health and Environmental Effects Research Laboratory, U.S. Environmental Protection Agency (EPA), Research Triangle Park, North Carolina, USA; 2 Center for Environmental Medicine, Asthma and Lung Biology, University of North Carolina, Chapel Hill, Chapel Hill, North Carolina, USA; 3 National Risk Management Research Laboratory, U.S. EPA, Research Triangle Park, North Carolina, USA; 4 Department of Environmental Modelling, Sensing and Analyses, Netherlands Organisation for Applied Scientific Research (TNO), Utrecht, the Netherlands

## Abstract

**Background:**

Emissions from solid fuels used for cooking cause ~4 million premature deaths per year. Advanced solid-fuel cookstoves are a potential solution, but they should be assessed by appropriate performance indicators, including biological effects.

**Objective:**

We evaluated two categories of solid-fuel cookstoves for eight pollutant and four mutagenicity emission factors, correlated the mutagenicity emission factors, and compared them to those of other combustion emissions.

**Methods:**

We burned red oak in a 3-stone fire (TSF), a natural-draft stove (NDS), and a forced-draft stove (FDS), and we combusted propane as a liquified petroleum gas control fuel. We determined emission factors based on useful energy (megajoules delivered, MJd) for carbon monoxide, nitrogen oxides (NOx), black carbon, methane, total hydrocarbons, 32 polycyclic aromatic hydrocarbons, PM2.5, levoglucosan (a wood-smoke marker), and mutagenicity in Salmonella.

**Results:**

With the exception of NOx, the emission factors per MJd were highly correlated (r ≥ 0.97); the correlation for NOx with the other emission factors was 0.58–0.76. Excluding NOx, the NDS and FDS reduced the emission factors an average of 68 and 92%, respectively, relative to the TSF. Nevertheless, the mutagenicity emission factor based on fuel energy used (MJthermal) for the most efficient stove (FDS) was between those of a large diesel bus engine and a small diesel generator.

**Conclusions:**

Both mutagenicity and pollutant emission factors may be informative for characterizing cookstove performance. However, mutagenicity emission factors may be especially useful for characterizing potential health effects and should be evaluated in relation to health outcomes in future research. An FDS operated as intended by the manufacturer is safer than a TSF, but without adequate ventilation, it will still result in poor indoor air quality.

**Citation:**

Mutlu E, Warren SH, Ebersviller SM, Kooter IM, Schmid JE, Dye JA, Linak WP, Gilmour MI, Jetter JJ, Higuchi M, DeMarini DM. 2016. Mutagenicity and pollutant emission factors of solid-fuel cookstoves: comparison with other combustion sources. Environ Health Perspect 124:974–982; http://dx.doi.org/10.1289/ehp.1509852

## Introduction

Although humans may have harnessed the power of fire as long as 1 million years ago (Berna et al. 2012), approximately 40% of us still cook and heat with fire in ways almost indistinguishable from those of our distant ancestors [International Agency for Research on Cancer (IARC) 2010]. Biomass in the form of various solid fuels (wood, straw, dung, charcoal, biomass briquettes, etc.) has yet to be replaced by modern energy carriers such as electricity and natural/petroleum gas for an estimated 2.8 billion people (Bonjour et al. 2013). In the United States, 500,000–600,000 low-income residents are estimated to be exposed to hazardous emissions from burning solid fuels inside their homes (Rogalsky et al. 2014). Smith et al. (2014) concluded that in 2010, household air pollution was responsible for 3.9 million premature deaths and ~4.8% of lost healthy life years, making it the most important environmental risk factor globally.

These deaths are due to a variety of diseases, including cardiovascular disease (Smith and Peel 2010), chronic obstructive pulmonary disease (Kurmi et al. 2010), low birth weight and stillbirth (Pope et al. 2010), upper-respiratory infections in children (Dherani et al. 2008), and lung cancer (IARC 2010). Household air pollution affects women disproportionately and is the second-highest risk factor in terms of the global burden of disease for women (Lim et al. 2012).

Global efforts are underway to deliver electricity and gas to underserved populations to replace solid-fuel cookstoves (Pachauri et al. 2013) because this is the ultimate step to improve health outcomes (Balakrishnan et al. 2014; Pachauri et al. 2013; Smith 2014). An interim approach involves introducing solid-fuel cookstoves with new designs that are safer than those currently in use. Thus, the World Health Organization (WHO) (2014) has developed air-quality guidelines by which cookstoves can be evaluated, and the International Organization for Standardization (ISO) (2012) established an International Workshop Agreement to provide interim guidelines for evaluating cookstoves on four performance indicators: *a*) fuel efficiency, *b*) total emissions, *c*) indoor emissions, and *d*) safety (from burns and injuries). The indoor-emissions indicator is related to WHO guidelines for air quality, but a specific performance indicator was not included for health effects.

Approximately 20 studies (Jetter et al. 2012; Just et al. 2013; Preble et al. 2014) have assessed many cookstoves burning a variety of solid fuels in the field and in the laboratory for multiple performance measures. Based on an analysis of 22 cookstoves, Jetter et al. (2012) suggested some metrics that could be used for setting international standards for ranking cookstove performance. These metrics included emissions of carbon monoxide (CO) and of particulate matter with an aerodynamic diameter ≤ 2.5 μm (PM_2.5_) based on useful energy delivered to the contents of the cooking vessel.

**Figure 1:**
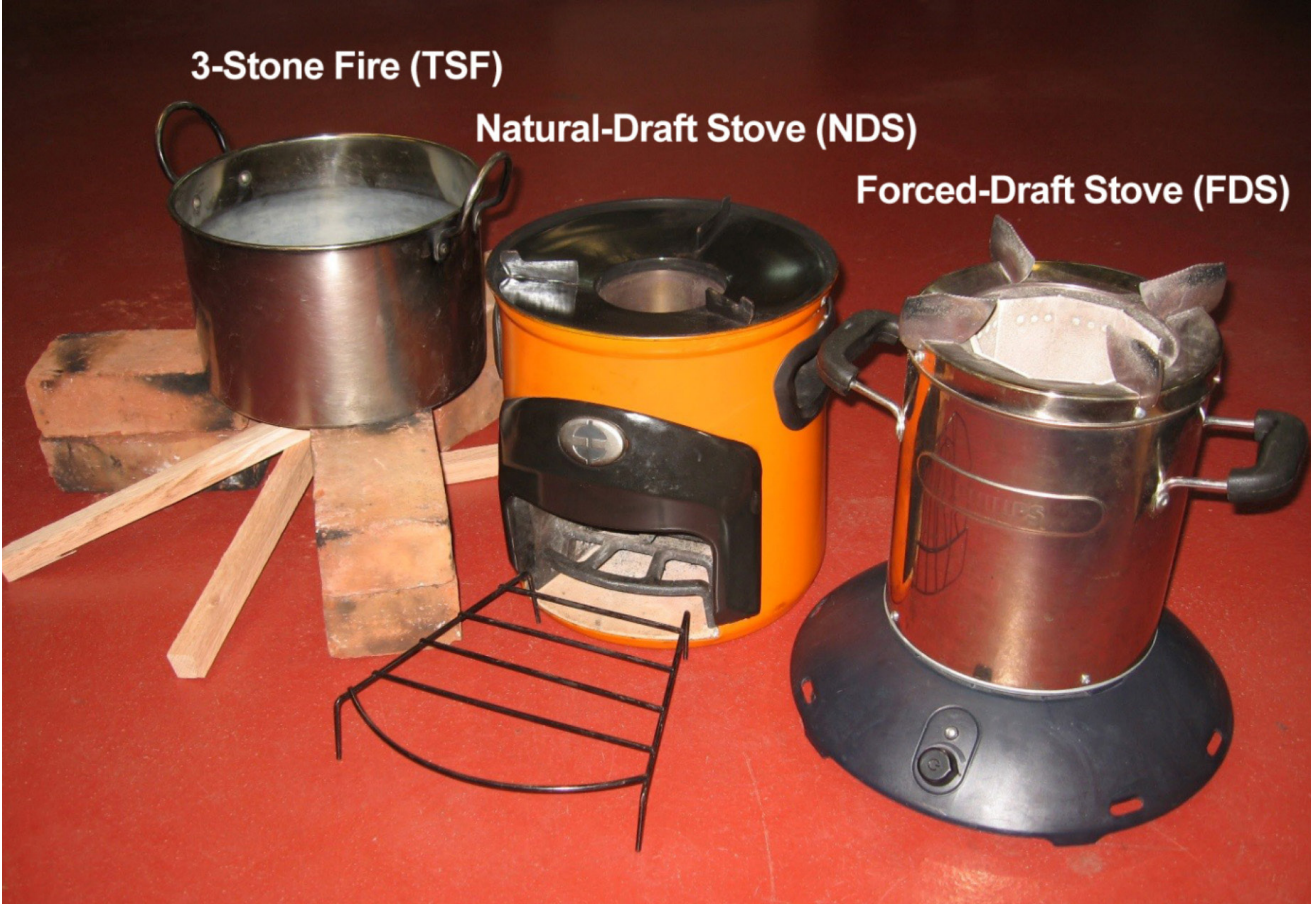
From left to right: a 3-stone fire (TSF), an Envirofit Model G-3300 natural-draft stove (NDS) or rocket-type stove, and a Philips HD4012 forced-draft stove (FDS).

However, an important question is whether these indicator pollutants provide an adequate indication of the health effects of the stoves. To help address this issue, we have evaluated two cookstoves representing two general categories of solid-fuel cookstoves: a natural-draft stove (NDS) and a forced-draft stove (FDS) (Figure 1). The rocket-type NDS is a vertical tube with an opening at the bottom to introduce the fuel, permitting air to flow by natural convection through the combustion chamber. In contrast, the FDS contains a fan in its base that forces air into the combustion chamber. We burned low-moisture red oak of the size prescribed by the manufacturers of these stoves under laboratory conditions similar to those used by Jetter et al. (2012) and in a 3-stone fire (TSF) for comparison. We also characterized the emissions from a propane stove as an example of a clean-burning liquified petroleum (LP) gas fuel.

We extended the assessments of Jetter et al. (2012) to also include black carbon (BC), and we also evaluated dichloromethane (DCM) extracts of the PM_2.5_ for the concentrations of 32 polycyclic aromatic hydrocarbons (PAHs) and levoglucosan (a marker of wood smoke), as well as for mutagenicity in *Salmonella*. We expressed all results as emission factors, correlated them, determined the percent reduction of the emission factors by the two stoves relative to those of a TSF, and compared the mutagenicity emission factors to those of other combustion emissions, some of which are associated with health effects, to provide an indication of the relative health impact of the stoves.

## Methods

### Combustion Conditions

Details of the combustion conditions, sources of material, and collection of PM_2.5_ have been described by Jetter et al. (2012). In brief, we burned *Quercus rubra* (red oak) with a moisture content of 6% as fuelwood in a TSF, in an Envirofit Model G-3300 NDS, and in a Philips HD4012 FDS (Figure 1). We used a modified water-boiling test protocol (WBT, version 4.2.3) (Global Alliance for Clean Cookstoves 2014) for all tests that consisted of *a*) high power to bring 5 L of water from ambient to boiling temperature and *b*) low power to maintain the water temperature at 3°C below boiling temperature for 45 min, and we used a standard 7-L cooking pot for all tests (Jetter and Kariher 2009; Jetter et al. 2012). We conducted up to four independent burns to assess reproducibility.

The size of the fuelwood was 2 cm × 2 cm × 36 cm for the TSF and NDS and 1.5 cm × 1.5 cm × 10 cm for the FDS; these sizes are typically used and were recommended by the manufacturers. The fuel sizes were matched to the stoves, and the comparisons we made were between cookstove systems consisting of the combination of stove, fuel, and operating procedure. Unlike our previous study (Jetter et al. 2012), we tested the NDS without a pot skirt because this configuration is considered more typical of stove use in the field (Adkins et al. 2010). As a reference, we also combusted propane gas in a Mikachi MNSS 1155 stove under the same conditions as described above.

### Collection of Emissions and Chemical Analyses

We collected emissions with a hood and dilution-tunnel system similar to that used by Jetter et al. (2012). Briefly, we collected integrated filter samples of PM_2.5_ on 47-mm diameter, 2-μm thick Teflon® filters that were weighed with a microbalance in an environmentally controlled chamber before and after collection. Sampling spanned two complete test phases (high-power followed by low-power operation), but we omitted the period between test phases to avoid any emissions released when the fire was extinguished before restarting for the second phase.

### Emission Factors

Emission factors based on useful energy delivered have denominators with units of megajoules delivered (MJ_d_), and those based on fuel energy used have denominators with units of megajoules thermal (MJ_th_). Energy efficiency is the ratio of useful-energy delivered to fuel energy used (MJ_d_/MJ_th_). We calculated pollutant and mutagenicity emission factors in a variety of units, and emission factors based on useful cooking energy (MJ_d_) enabled comparisons among all cookstove/fuel combinations. For example, we estimated for mutagenicity emission factors the number of revertants (rev) per MJ_d_ as: rev/MJ_d_ = (rev/mg PM_2.5_) × (mg PM_2.5_/MJ_d_).

We determined the cooking energy delivered (MJ_d_) from the *a*) sensible heat that raised the pot water temperature and *b*) latent heat that produced steam as described by Jetter et al. (2012). We calculated fuel energy used (MJ_th_) from the *a*) mass of fuel used and *b*) fuel-energy content measured with a bomb calorimeter; this value was 18,310 kJ/kg on a dry basis for the lower heating value. We calculated pollutant emission factors from weighted averages of values measured during high- and low-power operation. These weighted averages were directly comparable with the emission factors determined from the organic extracts of the PM because we combined filters from both power conditions to have suitable amounts of extract for chemical and mutagenicity analyses. For example, the CO per fuel-energy emission factors were calculated using the following equation:


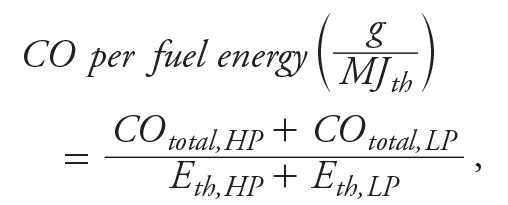


where

*CO_total,HP_* Mass of CO emitted during the high-power phase (grams)
*CO_total,LP_* Mass of CO emitted during the low-power phase (grams)
*E_th,HP_* Energy contained in the fuel burned during the high-power phase (megajoules)
*E_th,LP_* Energy contained in the fuel burned during the low-power phase (megajoules).

We measured a variety of pollutants in the emissions in real time, including CO (measured using a non-dispersive infrared analyzer), total hydrocarbons (THC) and methane (CH_4_) (measured using flame-ionization detection analyzers), nitrogen oxides (NO_X_) (measured using a chemiluminescence analyzer), and BC (measured using an aethalometer). We extracted the organics from the PM_2.5_ with dichloromethane (DCM), determined the percentage of extractable organic material (%EOM) by gravimetric analysis (DeMarini et al. 2004), and determined the concentrations in the extracts of 32 PAHs as described by Kooter et al. (2011) and the concentration of levoglucosan as described by Jedynska et al. (2015). We solvent-exchanged the extracts into dimethyl sulfoxide (DMSO) at 2 mg EOM/mL DMSO for the mutagenicity assays.

### Mutagenicity Assays

We performed a *Salmonella* plate-incorporation mutagenicity assay as described by Maron and Ames (1983) using strains TA98, TA100, TA104, and YG1041 ± metabolic activation (S9 mix). We used 1 mg of S9 protein/plate with Aroclor-induced Sprague Dawley rat liver S9 from Molecular Toxocology Inc. and incubated the plates for 3 days. Additional details of the procedures and supplies are described in Mutlu et al. (2013). The negative control was DMSO, and the positive controls were 2-nitrofluorene (3 μg/plate) for TA98 – S9 and YG1041 –S9; sodium azide (3 μg/plate) for strain TA100 – S9; 2-aminoanthracene (0.5 μg/plate) for TA98 + S9, TA100 + S9, YG1041 + S9, and TA104 + S9; and methylglyoxal (50 μg/plate) for strain TA104 – S9.

We defined a positive mutagenic response as a reproducible, dose-related response with a 2-fold or greater increase in revertants (rev) per plate relative to the DMSO control. Linear regressions were calculated over the linear portion of the dose–response curves, and the linear portion was defined by the line with the highest *r* ^2^ value. We deleted any dose that caused a down-turn in the curve and reduced the *r* ^2^ value relative to that produced by inclusion of the lower doses.

Strains TA98 and YG1041 detect mutagens that cause frameshift mutations, and strain YG1041 has enhanced expression of nitroreductase and acetyltransferase to increase the detection of mutagenic nitroarenes and aromatic amines, respectively. Strains TA100 and TA104 detect mutagens that induce base-substitution mutations, and strain TA104 detects oxidative mutagens because some of the targets for reversion in this strain are AT base-pairs. With some exceptions caused by limited sample quantity, we performed all experiments at one plate/dose in at least two independent experiments.

### Calculation of Mutagenicity Emission Factors

We calculated mutagenicity emission factors in the same way we calculated the pollution-emission factors described above. The mutagenic potencies (revertants/microgram EOM) were first calculated as the slope of the linear portion of the dose–response curves by averaging the data (revertants/plate for each dose) from at least two independent experiments. These mutagenic potencies (revertants/microgram EOM) were then multiplied by the %EOM (i.e., the EOM fraction, which is the micrograms EOM/microgram PM) to give the mutagenic potencies of the mass of particles (revertants/microgram particles), which is the same as revertants/microgram PM_2.5_. These values were multiplied by 1,000 to convert them to revertants/milligram PM_2.5_. The resulting mutagenic potencies of the particles (revertants/milligram PM_2.5_) were then converted to units of revertants/MJ_th_, rev/MJ_d_, revertants/kilogram fuel, and rev/hr using the measured PM_2.5_-emission factors reported herein. These reported values were in units of milligrams PM_2.5_/MJ_th_, milligrams PM_2.5_/MJ_d_, milligrams PM_2.5_/kilogram fuel, and milligrams PM_2.5_/hr. For example, revertants/milligram particles × milligram particles/MJ_th_ = revertants/MJ_th_.

### Statistical Analyses

We determined the mutagenic potencies of the EOM, of the PM_2.5_, and the mutagenicity emission factors as follows. We calculated the slope and standard error (SE) from the raw mutagenicity data (revertants/plate) to determine the mutagenic potency of the EOM, expressed as rev/μg EOM ± SE; we performed this calculation using regression models with SAS Proc GLM (SAS Institute Inc.). We multiplied these mutagenic potencies by 1,000 to convert them to revertants/milligram EOM ± SE, and then we multiplied these by the %EOM, which was a constant, to determine the mutagenic potencies of the PM_2.5_ (revertants/milligram PM_2.5_ ± SE). We then converted the revertants/milligram PM_2.5_ to a mutagenicity emission factor by using the appropriate PM_2.5_ emission factor (milligrams PM_2.5_/MJ_th_, milligrams PM_2.5_/MJ_d_, milligrams PM_2.5_/kilogram fuel, or milligrams PM_2.5_/hour) for each stove. These PM_2.5_-emission factors were associated with an SE; thus, we calculated the SE for the mutagenicity emission factors according to Goodman’s expression, using the formula s^2^_AB_ = B^2^s^2^_A_ + A^2^s^2^_B_ + s^2^_A_ s^2^_B._ We performed tests for differences among the FDS, NDS, TSF, and diesel engine mutagenicity emission factors using Wald statistical tests for each relevant pair. The FDS, the NDS, the TSF, and the diesel engine were considered to be independent; thus, pairwise covariances were assumed to be 0. We calculated Pearson correlation coefficients to compare all of the emission factors. We considered results significantly different for *p* < 0.05.

We performed two independent combustion experiments involving mutagenicity measurements for the FDS. Consequently, we performed statistical analyses to compare the variability of five pollutant and eight mutagenicity emission factors (all expressed per MJ_d_) between these replicate experiments. For this analysis, we reported the experimental parameters and pollutant emission factors as weighted averages (described in the subsection entitled “Emission factors” in “Methods”) with sample standard deviations (SDs), standard errors (SEs), coefficients of variation (CVs), and number of replicates (*n*s). Among these factors, carbon dioxide (CO_2_) was the primary product of biomass combustion. We used the CO_2_ emission factor and the rate at which fuel was consumed (the fuel-burn rate) to compare the reproducibility of the two experiments. We did not use emissions data from Experiment 1 to generate any of the emission factors or mutagenicity data reported elsewhere in the manuscript. Instead, these were included in the statistical analysis only for the purpose of comparing replicate runs of the FDS. Experiment 2 represents the set of replicate experiments using the FDS that we analyzed in this study and report in this paper.

We compared the emission factors between the two FDS experiments described above using a two-tailed Student’s *t*-test with Welch’s correction. We compared the mutagenicity data using linear regression for the dose–response curves generated by two independent mutagenicity measurements for each of two combustion experiments for a total of two experiments. We then compared the slopes of the resulting regressions by using Statgraphics Centurion XVI (Statpoint Technologies, Inc.). The Comparison of Regression Lines procedure is designed to compare the regression lines relating Y and X at two or more levels of a categorical factor. Tests were performed to determine whether there were significant differences between the intercepts and the slopes at the different levels of that factor. We plotted the regression lines, identified unusual residuals, and made predictions using the fitting model. We analyzed the data from the two groups in a multiple-regression model that allowed for a separate intercept and slope for each group. We used Student’s *t*-test within the model to test for a difference between the two slopes. We considered results significantly different for *p* < 0.05.

## Results

### Pollutant Emission Factors

Weighted averages for high- and low-power test conditions of the pollutant emission factors determined from continuous-emission monitors as well as from the integrated PM_2.5_, which was collected on filters, are shown in Table 1, and measured parameters from the two individual cooking-power conditions are shown in Table S1. Our results for CO and PM_2.5_ confirmed prior analyses of the NDS and FDS (Jetter et al. 2012). The emissions ranked as follows based on these pollutant emission factors: Propane < FDS < NDS < TSF; the energy efficiencies (100 × MJ_d_/MJ_th_) for these technologies were 67, 36, 32, and 24%, respectively.

**Table 1:** Pollutant emission factors expressed as weighted averages for two power levels.

Pollutant/emission factor	Emission source^*a*^			
	TSF	NDS	FDS	Propane
PM_2.5_
mg/MJ_d_	449.4	198.6	78.3	1.2
mg/MJ_th_	106.8	64.0	28.4	0.8
mg/kg of fuel	1544.9	920.7	454.2	35.2
mg/hr	1396.5	770.2	273.0	4.8
CO
g/MJ_d_	11.8	4.4	1.2	0.2
g/MJ_th_	2.7	1.3	0.4	0.1
g/kg of fuel	40.6	20.5	7.2	5.8
g/hr	36.7	17.0	4.2	0.8
THC
g/MJ_d_	1.1	0.4	0.1	0.0
g/MJ_th_	0.3	0.1	0.0	0.0
g/kg of fuel	3.9	2.1	0.7	0.6
g/hr	3.5	1.7	0.4	0.1
CH_4_
g/MJ_d_	0.3	0.1	0.0	0.0
g/MJ_th_	0.1	0.0	0.0	0.0
g/kg of fuel	1.1	0.4	0.2	0.0
g/hr	0.9	0.3	0.1	0.0
NO_x_
g/MJ_d_	0.2	0.2	0.1	0.1
g/MJ_th_	0.0	0.0	0.0	0.0
g/kg of fuel	0.6	0.7	0.6	1.4
g/hr	0.6	0.6	0.4	0.2
BC
mg/MJ_d_	254.0	68.0	42.3	0.0
mg/MJ_th_	62.7	22.0	15.1	0.0
mg/kg of fuel	868.4	320.2	247.5	0.0
mg/hr	786.2	269.3	146.4	0.0
Abbreviations: BC, black carbon; CH_4_, methane; CO, carbon monoxide; FDS, forced-draft stove; MJ_d_, megajoules energy delivered to the cooking pot; MJ_th_, megajoules thermal energy; NDS, natural-draft stove; NO_x_, oxides of nitrogen; PM_2.5_, particulate material ≤ 2.5 μm in diameter; THC, total hydrocarbons; TSF, three-stone fire. ^***a***^Data for PM_2.5_ derived from samples collected on filters; all other data derived from continuous-emission monitoring.				

**Figure 2:**
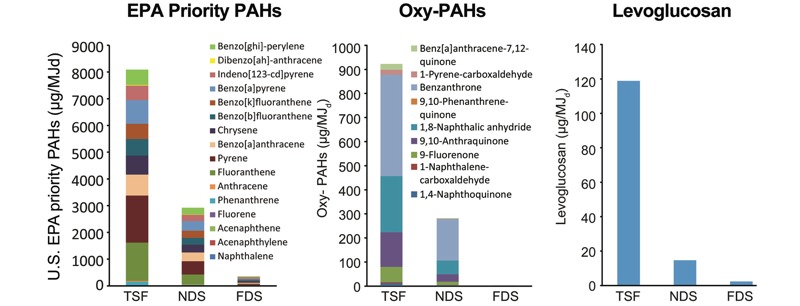
Pollutant emission factors expressed as micrograms/megajoules energy delivered to the cooking pot (MJ_d_) for the 16 U.S. EPA priority poloycyclic aromatic hydrocarbons (PAHs), the 9 oxy-PAHs, and levoglucosan; data are from Table S2. Abbreviations: FDS, forced-draft stove; NDS, natural-draft stove; TSF, three-stone fire.

Gravimetric analyses of the DCM extracts of the PM collected on the filters showed that the %EOMs were 33.6% for TSF, 17.9% for NDS, 3.0% for FDS, and 0% for propane gas. Because there were no detectable levels of EOM in the propane emissions, we did not subject that extract to chemical analysis. However, analyses of the other extracts resulted in chemical-emission factors for the 16 U.S. Environmental Protection Agency (EPA) priority PAHs, the 9 oxy-PAHs, and levoglucosan, as shown in Table S2. Although we also analyzed these extracts for 6 nitro-PAHs as previously described (Mutlu et al. 2013), we did not find detectable levels of any of them (data not shown). Using data from Table S2, Figure 2 shows that for these PAH-emission factors, the emissions ranked as follows: FDS < NDS < TSF.

### Mutagenic Potencies of EOM and Particles

**Figure 3:**
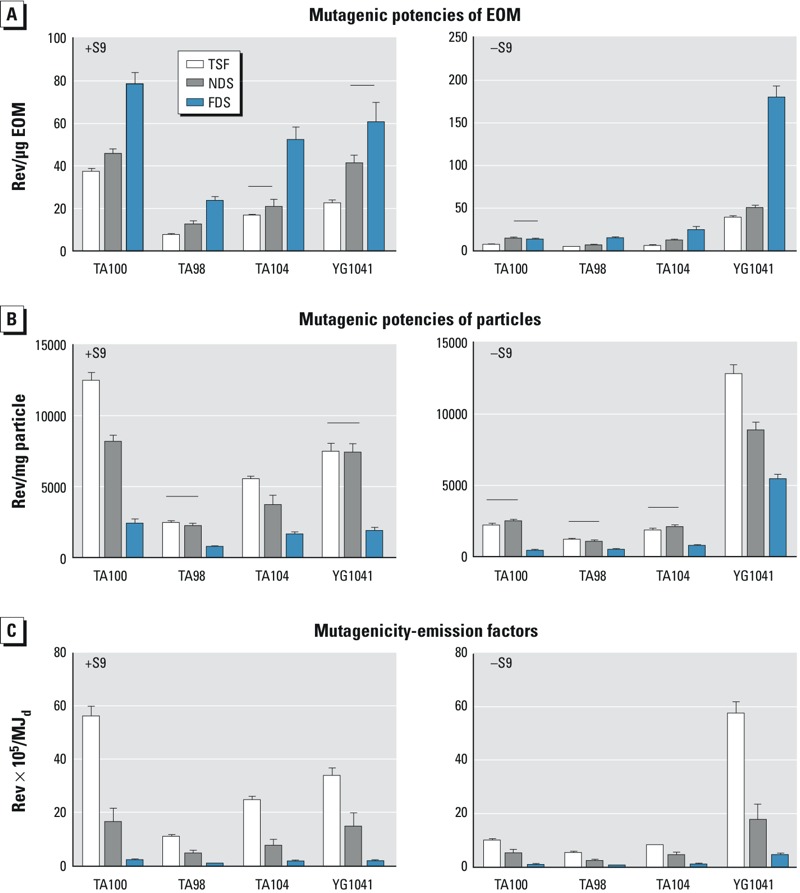
Mutagenic potencies of the (*A*) extractable organic material (EOM) and (*B*) particles; data from Tables S4 and S5. (*C*) Mutagenicity emission factors; data from Table 2. These values were calculated as described according to the formulas in “Methods” under the subheading, “Calculation of mutagenicity emission factors*.”* The calculation involved the determination of the slope of the linear portion of the dose–response curve created by the average of the primary data (revertants/plate) from at least two independent mutagenicity experiments. These slopes (revertants/microgram EOM) were then multiplied by the %EOM to give revertants/microgram particles, which were then multiplied by 1,000 to give revertants/milligram particles, which is the same as revertants/milligram PM_2.5_. The revertants/milligram PM_2.5_ values were then converted to mutagenicity emission factors [revertants/megajoules energy delivered to the cooking pot (MJ_d_)] by multiplying them by PM_2.5_ emission factors as described in the subsection of “Methods” entitled “Statistics” and using the following PM_2.5_ emission factors: milligrams PM_2.5_/megajoules thermal energy (MJ_th_), milligrams PM_2.5_/MJ_d_, milligrams PM_2.5_/kilogram fuel, or milligrams PM_2.5_/hour, which were determined as described in the “Methods” subsection entitled “Emission factors.” The resulting mutagenicity emission factors are presented in Table 2. All mutagenicity emission factors were derived from positive mutagenic potency data (revertants/microgram EOM); that is, the dose–response reached or exceeded a 2-fold increase in revertants/plate relative to the dimethyl sulfoxide (DMSO) control. The standard error (SE) is shown for each histogram, and the horizontal bars represent comparisons between emissions where the *p*-values were > 0.05 and, thus, not significantly different; all other comparisons of the three emissions within a strain/S9 condition were significantly different from each other (*p* < 0.001).

There was no detectable EOM in the extract of the propane emissions, and the extract was not mutagenic (data not shown); the average revertants/plate for the three emissions are shown in Table S3. With only a few exceptions, the mutagenic potencies of the EOM ranked as follows among the strain/S9 combinations: TSF < NDS < FDS (Figure 3A and Table S4 for corresponding numeric data). The highest mutagenic potencies were in strain TA100 + S9, which detects PAHs, and strain YG1041 – S9, which detects nitroarenes.

In contrast to the EOM, the mutagenic potencies of the particles generally ranked in the opposite order as that of the EOM: FDS < NDS < TSF (Figure 3B and Table S5 for corresponding numeric data). However, the mutagenic potencies of the particles in five of the eight strain/S9 combinations were not significantly different between the TSF and the NDS (Figure 3B). A sequential decrease in the mutagenic potencies of the particles from all three emissions was found for TA100 + S9 (which detects PAHs), TA104 + S9 (which detects oxidative mutagens), and YG1041 – S9 (which detects nitroarenes). This general reversal of potencies among the stoves between EOM and particles indicates that high-efficiency combustion (FDS) produces organics that are more mutagenic per mass of EOM than does low-efficiency combustion (TSF). However, the particles from high-efficiency combustion (FDS) had only 10% of the amount of EOM compared with particles from low-efficiency combustion, resulting in particles from the FDS that were less mutagenic per unit mass than those from the TSF.

### Mutagenicity Emission Factors

Regardless of the units of expression, the strain, or the presence/absence of S9, the emissions ranked as follows based on revertants/MJ_d_: FDS < NDS < TSF (Table 2); the numerical values are shown in Figure 3. The revertants/MJ_d_ were significantly different (*p* < 0.001) among all three emissions within each strain/S9 combination (Figure 3). In the presence of S9, the largest mutagenicity emission factors for each emission expressed by any unit were in strain TA100 + S9, indicating an important role for S9-requiring base-substitution mutagens such as PAHs. The second was in YG1041, indicating a role for aromatic amines. The largest values in the absence of S9 were in strain YG1041, indicating an important role for frameshift mutagens such as nitroarenes. The results in strain TA104 with or without S9 indicated a role for oxidative mutagens. The lowest values were in strain TA98 ± S9, suggesting that frameshift mutagens in addition to or other than nitroarenes also contributed to the mutagenicity of the emissions. For all strains, the revertants/MJ_d_ were reduced by ~50% by the NDS and by > 90% by the FDS relative to the TSF (Table 2).

**Table 2:** Mutagenicity emission factors (× 10^5^) ± SE derived from organic extracts of particulate matter*^a^*.

Units/strain	TSF		NDS		FDS	
	+S9	–S9	+S9	–S9	+S9	–S9
Rev/MJ_th_
TA100	12.7 ± 0.9	2.2 ± 0.2	4.6 ± 0.6	1.4 ± 0.2	0.7 ± 0.2	0.2 ± 0.0
TA98	2.4 ± 0.2	1.2 ± 0.1	1.2 ± 0.2	0.6 ± 0.1	0.2 ± 0.0	0.1 ± 0.0
TA104	5.6 ± 0.4	1.8 ± 0.2	2.1 ± 0.5	1.2 ± 0.2	0.4 ± 0.1	0.2 ± 0.1
YG1041	7.6 ± 0.8	13.0 ± 1.0	4.2 ± 0.7	5.0 ± 0.7	0.5 ± 0.1	1.5 ± 0.4
Rev/MJ_d_
TA100	55.7 ± 4.2	9.6 ± 0.9	16.1 ± 5.7	4.9 ± 1.7	1.8 ± 0.4	0.3 ± 0.7
TA98	10.7 ± 0.9	5.1 ± 0.6	4.3 ± 1.6	2.0 ± 0.7	0.5 ± 0.01	0.3 ± 0.1
TA104	24.4 ± 1.7	7.9 ± 0.1	7.2 ± 2.9	4.1 ± 1.5	1.2 ± 0.3	0.6 ± 0.2
YG1041	33.3 ± 3.5	57.2 ± 4.5	14.5 ± 5.3	17.4 ± 6.2	1.4 ± 0.4	4.2 ± 1.0
Rev/kg fuel
TA100	191.4 ± 16.3	32.9 ± 3.4	74.4 ± 9.2	22.6 ± 2.8	10.6 ± 2.5	1.7 ± 0.4
TA98	36.8 ± 3.5	17.6 ± 2.3	20.0 ± 3.1	9.4 ± 1.2	3.2 ± 0.8	2.0 ± 0.5
TA104	84.0 ± 6.7	27.5 ± 3.7	33.6 ± 7.4	18.8 ± 2.5	7.1 ± 1.8	3.2 ± 0.9
YG1041	114.3 ± 12.7	196.7 ± 17.4	67.3 ± 9.7	80.8 ± 10.4	8.2 ± 2.3	24.4 ± 5.7
Rev/hr
TA100	173.0 ± 18.5	29.7 ± 3.6	62.2 ± 10.5	18.9 ± 3.2	6.4 ± 2.2	1.0 ± 0.4
TA98	33.2 ± 3.8	16.0 ± 2.3	16.7 ± 3.2	7.9 ± 1.3	1.9 ± 0.7	1.2 ± 0.4
TA104	76.0 ± 7.8	24.8 ± 3.7	28.1 ± 7.0	15.7 ± 2.8	4.2 ± 1.5	1.9 ± 0.7
YG1041	103.3 ± 13.1	177.8 ± 19.5	56.3 ± 10.4	67.6 ± 11.6	4.9 ± 1.9	14.6 ± 5.1
Abbreviations: FDS, forced-draft stove; MJ_d_, megajoules energy delivered to the cooking pot; MJ_th_, megajoules thermal energy; NDS, natural-draft stove; Rev, revertant; TSF, three-stone fire. ^***a***^These values were calculated as described according to the formulas in “Methods” under the subheading, *“*Calculation of mutagenicity emission factors*.”* The calculation involved determination of the slope of the linear portion of the dose–response curve produced by the average of the primary data (revertants/plate) from at least two independent mutagenicity experiments. These slopes [revertants/microgram extractable organic material (EOM)] were then multiplied by the %EOM to give revertants/microgram particles, which were then multiplied by 1,000 to give revertants/milligram particles, which is the same as revertants/milligram PM_2.5_. The revertants/milligram PM_2.5_ values were then multiplied as described in “Methods” (under the subheading,* “*Statistics”) by the following PM_2.5_-emission factors: milligrams PM_2.5_/MJ_th_, milligrams PM_2.5_/MJ_d_, milligrams PM_2.5_/kilogram fuel, or milligrams PM_2.5_/hour, which were determined as described in “Methods” under the subheading, “Emission factors.” The resulting mutagenicity emission factors are those presented in this table. All mutagenicity emission factors were calculated from mutagenic potency data (revertants/microgram EOM) that were positive; that is, the dose-response reached or exceeded a 2-fold increase in revertants/plate relative to the DMSO control.						

To determine the reproducibility of the pollutant emission factors, we calculated them from a set of multiple combustion experiments with the TSF, the NDS, the FDS, and propane fuel. The experiments were highly reproducible, as indicated by the coefficient of variation (CV) values < 10% for most fuel-burn rates and for CO_2_ emissions, the primary product of combustion (Tables S6 and S7). Numbers of replicates (*n*) are shown in the tables. The relatively low CV values (< 10%) for the fuel-burn rates and CO_2_ emission factors (expressed as either MJ_d_ or MJ_th_) indicate that the stoves were operated consistently across all test replicates, resulting in reproducible data. The factors with CV values greater than those of the fuel-burn rates and CO_2_ emission factors reflect the inherent variability of the fuel stock and physical processes involved in biomass combustion. With the exception of the emission factors for BC (*p* = 0.039) and mutagenicity in *Salmonella* strains TA98 – S9 (*p* < 0.001) and YG1041 – S9 (*p* = 0.013), there were no significant differences between replicate experiments for the other six emission factors for the FDS, including mutagenicity in TA98 + S9 and TA100 + S9 (see Table S8).

### Comparison of Emission Factors

With the exception of NO_x_ (Pearson correlations 0.58–0.76), the other emission factors were highly correlated (Pearson correlations ≥ 0.97) (see Table S9). Table 3 shows the percent reduction in the emission factors achieved by the two stoves relative to the TSF. Similar results were obtained for emission factors based on continuous-emission monitoring to those based on extracts of the PM_2.5_. Excluding NO_x_ and averaging the percent reduction of the remaining emission factors expressed per MJ_d_ in Table 3 (and considering that total PAHs includes the 16 U.S. EPA PAHs and the oxy-PAHs), the NDS and FDS reduced these emission factors by 68 and 92%, respectively, relative to the TSF.

**Table 3:** Reduction (%) in emission factors by the two stoves compared with the TSF.

Sample collection method	Emission factor (/MJ_d_)	Average reduction ± SE^*a*^ (%)	
		NDS	FDS
Integrated-filter sampling	mg PM_2.5_	55.8 ± 31.3	82.6 ± 7.1
Continuous-emission monitoring	g CO	62.7 ± 4.9	89.8 ± 1.1
	g THC	63.6 ± 17.9	90.9 ± 6.5
	g CH_4_	66.7 ± 10.4	100.0 ± 3.5
	mg BC	73.2 ± 7.6	83.3 ± 3.3
	g NO_x_	0.0^*c*^	50.0^*c*^
Extract of PM_2.5_ from filters	μg 16 U.S. EPA PAHs	63.9^*c*^	95.5^*c*^
	μg oxy-PAHs	69.4^*c*^	89.0^*c*^
	μg Total PAHs	64.4^*c*^	94.9^*c*^
	Rev × 10^5^ in TA100 + S9	72.1^*c*^	97.0^*c*^
	μg Levoglucosan	87.7^*c*^	98.0^*c*^
Average^*b*^		68.2	92.0
Abbreviations: BC, black carbon; CH_4_, methane; CO, carbon monoxide; FDS, forced-draft stove; MJ_d_, megajoule energy delivered to the cooking pot; NDS, natural-draft stove; NO_x_, oxides of nitrogen; PAH, polycyclic aromatic hydrocarbon; PM_2.5_, particulate material ≤ 2.5 μm in diameter; Rev, revertant; SE, standard error; THC, total hydrocarbons. ^***a***^The number of samples was generally four; the exceptions are noted in Table S5. ^***b***^Average of the percent reduction values omitting those for NO_x_, and considering that the total PAHs includes both the 16 U.S. EPA PAHs and the oxy-PAHs. ^***c***^Data insufficient to determine SE.			

## Discussion

### Pollutant Emission Factors

As noted in the “Materials” section, the comparisons we made were between cookstove systems consisting of a stove, the appropriate fuel, and the operating procedure for each stove. We operated the stove systems as intended by the manufacturers; performance may vary if the systems are not operated as intended. The TSF configuration and the thermal efficiency were similar to those in Jetter and Kariher (2009), but the fuel-burning rate was lower here because we used a larger-sized fuelwood that was more representative of the average size of fuelwood used in the field (Rosenbaum et al. 2013). The thermal efficiency for the NDS was 38.0% in our previous study and 31.2% here, and the high-power fuel-burning rate for the TSF was 25.4 g/min in our previous study and 15.3 g/min here (see Table S1). Our emission factors for CO and PM_2.5_ for the NDS were also similar to those obtained in our previous study (Jetter et al. 2012), but the thermal efficiency was reduced because in the present study, we tested the stove without a pot skirt, which is considered to be the typical mode of operation in the field. The FDS was tested with the same configuration and had nearly the same fuel-burning rates as those obtained in our previous study (Jetter et al. 2012), and the results were similar.

Emission rates for PM_2.5_ and CO may be used to rank stoves according to tiers of performance for indoor emissions as defined by the ISO (2012) guidelines: Tier 0 represents the lowest level of performance, typical for a TSF, whereas Tier 4 represents the level of performance of an LP/natural gas stove. The results show that many other types of emission factors (THC, CH_4_, BC, PAHs, levoglucosan, and mutagenicity) correlate with PM_2.5_ and CO (see Table S9) for the stove and fuel combinations tested under our controlled conditions. Relative to the TSF, the FDS reduced the emission factors associated with products of incomplete combustion by > 80% (Table 3); the FDS is rated in Tier 3 for indoor emissions, with PM_2.5_ ≤ 480 mg/hr (Jetter et al. 2012).

Other studies have measured as many as 38 or 48 PAHs, along with 3 other pollutant emission factors (organic carbon, PM, elemental carbon) for various fuels and stoves (Shen et al. 2012, 2013). Here, we determined the emission factors for only 32 PAHs, but we also determined the emission factors for 6 other pollutants, a marker of woodsmoke (levoglucosan), and for the first time, we determined the mutagenicity for an NDS, an FDS, and a TSF for comparison purposes. Improved combustion efficiency was associated with reduced emission factors for the various species of PAHs, levoglucosan, and mutagenicity. The high correlations among all of the emission factors other than NO_x_ were expected because these indicators are all measures of the incomplete combustion of carbon species. In contrast, NO_x_ is associated with fuel and atmospheric nitrogen and results from complex mechanisms related to flame aerodynamics, fuel-oxidant mixing, and temperature; NO_x_ is associated only indirectly with carbon burnout during combustion.

### Mutagenicity Emission Factors

**Figure 4:**
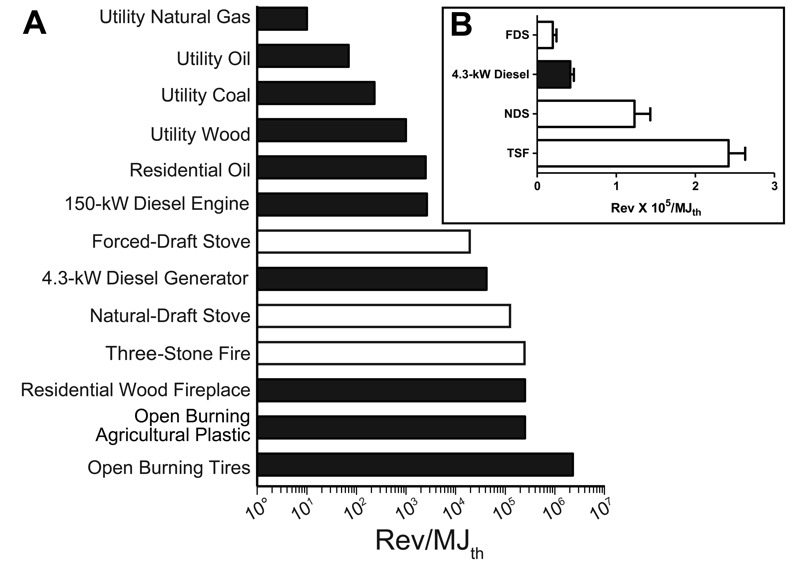
(*A*) Comparison of mutagenicity emission factors in strain TA98 + S9 [revertants/megajoules thermal energy (MJ_th_)] for a variety of combustion emissions. (*B*) Data for the four emissions for which standard error (SE) values were available, replotted on a linear scale. Data for diesel engines are from Mutlu et al. (2015); data for remaining black bars are from DeMarini et al. (1992, 1994). Data for white bars are from Table 2. We calculated the mutagenicity emission factor for the 150-kW diesel engine from data in Turrio-Baldassarri et al. (2004) and first published that value (2.76 × 10^3^ rev/MJ_th_ in TA98 + S9) in Mutlu et al. (2015). We calculated the SE for the 4.3-kW diesel generator using data from Mutlu et al. (2015) by the same method we used here to calculate the SE for the 3 stoves. The revertants × 10^5^/MJ_th_ in TA98 + S9 for values with a calculated SE were 0.42 ± 0.04 for the 4.3-kW diesel generator, 0.20 ± 0.04 for the forced-draft stove (FDS), 1.23 ± 0.20 for the natural-draft stove (NDS), and 2.42 ± 0.21 for the three-stone fire (TSF). Comparisons among these four emissions showed them to be significantly different from each other (*p* < 0.001).

Figure 4A summarizes the published mutagenicity emission factors (revertants/MJ_th_) in strain TA98 + S9 for a range of combustion emissions, including those from the present study. Data for the cookstoves were expressed as thermal energy (Table 3) rather than as delivered energy to permit comparisons because the other emissions were expressed per unit of fuel energy used. The revertants/MJ_th_ values were lower for fossil fuels burned in large power-conversion systems employing engineering principles designed to maximize temperatures, fuel and oxidant mixing, flame stability, and combustion efficiency, and they were higher in smaller, less-efficient systems and open, uncontrolled combustion of poorly characterized fuels. Gaseous, homogeneous fuels produced lower emission factors than solid, heterogeneous fuels, as also illustrated by our data in Tables S5 and S6 for wood versus propane.

For the four emissions for which SE values were known (the diesel generator and the three stoves), all had revertants/MJ_th_ values that were significantly different from each other (*p* < 0.001) (Figure 4B). The mutagenicity emission factor of the most efficient stove (FDS) was significantly lower than that of the 4.3-kW diesel generator, but it was an order of magnitude greater than that of the 150-kW diesel engine. Thus, the FDS was in between a large diesel engine (from a bus) and a small diesel generator. The revertants × 10^5^/MJ_th_ values were 0.0276, 0.2, 0.42, 1.23, and 2.42/2.5/2.5 for the diesel engine, the FDS, the diesel generator, the NDS, and the TSF/residential wood fireplace/open burning of agricultural plastic, respectively (Figure 4).

The pollutant emission factors associated with the propane stove were generally orders of magnitude lower than those associated with biomass burning (Table 1). Similarly, the mutagenicity emission factor in TA98 + S9 based on fuel energy used (revertants/MJ_th_) for utility natural gas (20 rev/MJ_th_) (DeMarini et al. 1992) is three orders of magnitude lower than that of the best stove in the present analysis (FDS at 20,000 rev/MJ_th_) (Table 2). Such results indicate that emissions from the combustion of liquid/gas fuels are considerably less polluting than those from the solid-fuel stoves that we evaluated here.

Consistent with this observation, a study of pregnant Peruvian women reported that 12 women who cooked with wood and 4 women who cooked with kerosene had higher urinary concentrations of hydroxylated PAHs (1-, 2-, and 3-hydroxyfluorene and 2- and 4-hydroxyphenanthrene) than 27 women who cooked with LP and gas fuels (Adetona et al. 2013a). Such data indicate the importance of substituting wood or kerosene with LP/gas fuels or electricity, improving ventilation, and/or developing cleaner solid-fuel technologies to replace solid-fuel biomass burning for cooking among underserved populations (Balakrishnan et al. 2014; Pachauri et al. 2013).

The greatest reduction in mutagenicity emission factors was by the FDS relative to the TSF in TA100 + S9 (Figure 3), which detects PAH mutagenicity, consistent with the reductions in PAH-emission factors by the FDS relative to the TSF (Figure 2). Urinary mutagenicity was elevated in 49 Brazilian charcoal workers with high exposure to woodsmoke outdoors compared with 34 workers with no exposure, and this correlated with urinary concentrations of 2-naphthol and 1-pyrenol, of which the parent compounds (naphthalene and pyrene) have been found in woodsmoke (Kato et al. 2004). A study by Long et al. (2014) of Mayan individuals that used traditional wood-fired steam baths reported that urinary mutagenicity and exhaled CO both increased after use of the steam bath and were significantly correlated (*r* ^2^ = 0.53, *p* < 0.001).

Using data for all three emissions (TSF, NDS, FDS), we regressed values of revertants of TA100 + S9/MJ_d_ (55.7, 16.1, and 1.8 rev × 10^5^/MJ_d_, respectively) (Table 2) against micrograms oxy-PAHs/MJ_d_ (921.9, 282.1, and 101.1 μg/MJ_d_, respectively) (see Table S2) and estimated a slope of 0.06 × 10^5^ rev/μg oxy-PAHs, which was approximately nine times greater than the corresponding estimate for TA100 + S9/MJ_d_ and the 16 U.S. EPA priority PAHs (0.007 × 10^5^ rev/μg U.S. EPA PAHs). The higher mutagenic potency (slope) of the oxy-PAHs further supports the contribution of the oxy-PAHs to the mutagenicity of these emissions.

This finding is consistent with studies showing that oxy-PAHs are associated with fine PM and cookstove combustion (Shen et al. 2011). In addition, oxidative damage was increased in human lung epithelial and monocytic cell lines exposed *in vitro* to ambient PM from a Danish village with high wood-stove use and to PM collected from wood-stove exhaust (Danielsen et al. 2011). Using urinary concentrations of 8-isoprostane (a product of lipid peroxidation) as a biomarker of oxidative stress, Commodore et al. (2013) found an increase in this biomarker in samples of 48-hr personal PM_2.5_ exposures among 69 Peruvian women using wood for cooking indoors. Adetona et al. (2013b) found a pre- versus post-shift increase in 8-hydroxy-2´-deoxyguanosine (8-OH-dG) in 3 wildland firefighters with 0–2 years of firefighting experience; 8-OH-dG was also associated with years of firefighting among 17 subjects. Thus, it appears that chronic exposure to woodsmoke elevates oxidative stress biomarkers.

### Issues Associated with Introducing Advanced Cookstoves

Although the FDS evaluated here has been adopted and used more than another type of FDS (the Oorja) in field studies in India (Mukhopadhyay et al. 2012), systematic reviews have shown that more work is needed for the successful introduction of improved fuels and/or cookstoves (Lewis and Pattanayak 2012; Rehfuess et al. 2014). More than a dozen studies have evaluated indoor-air quality or the health of the residents after interventions involving stoves or chimneys. Although some of the studies reported significant improvements in either the indoor-air quality (Fitzgerald et al. 2012; Noonan et al. 2012) or in biomarkers (e.g., carboxyhemoglobin, exhaled CO, lymphocyte DNA damage, urinary 1-hydroxypyrene, or ST-segment depression) in the residents of the home (Eppler et al. 2013; McCracken et al. 2011; Torres-Dosal et al. 2008) after the intervention, many reported either no improvements or, at best, modest improvements in health outcomes. For example, some of the studies showed that although urinary concentrations of 10 hydroxylated metabolites of naphthalene, fluorene, phenanthrene, and pyrene were reduced, the mean concentrations of some compounds, such as 1-hydroxypyrene, remained in the 95th percentile of the U.S. population based on the National Health and Nutrition Survey 2001–2002 or similar to that of smokers (Li et al. 2011; Riojas-Rodriguez et al. 2011). Installation of chimneys in rural homes in Peru or Guatemala to improve indoor air quality or health outcomes did not result in significant reductions in the frequency of childhood pneumonia (Smith et al. 2011) or in concentrations of CO and PM_2.5_ in the home (Hartinger et al. 2013; Pollard et al. 2014).

### Conclusions

To our knowledge, our data are the first to link pollutant and mutagenicity emission factors for solid-fuel cookstoves of these types and to compare the mutagenicity emission factors to those of other combustion emissions. Many emission factors (PAHs, PM_2.5_, THC, CO, CH_4_, BC, levoglucosan, and mutagenicity) were highly correlated for the stove and fuel combinations tested under our controlled conditions. Based on fuel-energy used (MJ_th_), the mutagenicity emission factor for the most efficient stove (the FDS) was between those of a large diesel bus engine and a small diesel generator (Figure 4). Not surprisingly, the mutageniciy emission factor of the TSF was similar to that of other open burning such as residential wood fireplaces and open burning of agricultural plastic (Figure 4). We conclude that without adequate ventilation, even the FDS would result in exposure to highly mutagenic emissions indoors, resulting in poor indoor air quality.

Ultimately, the introduction and reliable supply of electricity and LP/natural gas fuels are essential for making significant improvements in health outcomes for the billions of people who currently have limited or no access to such energy sources for cooking (Pachauri et al. 2013; Smith 2014). While the infrastructure is being developed to supply electricity and LP/natural gas fuels for this population, the introduction of advanced, solid-fuel, vented cookstoves is critical to meeting their intermediate needs (Anenberg et al. 2013).

## Supplemental Material

(470 KB) PDF
