# Supplementary material for: Mutagenicity and Pollutant Emission Factors of Solid-Fuel Cookstoves: Comparison with Other Combustion Sources
Source: Environ Health Perspect. 2016 Feb 19;124(7):974–82. doi: 10.1289/ehp.1509852 (PMC4937857; doi:10.1289/ehp.1509852)
Supplement: (470 KB) PDF [file ehp.1509852.s001.acco.pdf]

Environ Health Perspect
DOI: 10.1289/ehp.1509852

Note to readers with disabilities: *EHP* strives to ensure that all journal content is accessible to all readers. However, some figures and Supplemental Material published in *EHP* articles may not conform to 508 standards due to the complexity of the information being presented. If you need assistance accessing journal content, please contact ehp508@niehs.nih.gov. Our staff will work with you to assess and meet your accessibility needs within 3 working days.

Supplemental Material

Mutagenicity- and Pollutant-Emission Factors of Solid-Fuel Cookstoves: Comparison to Other Combustion Sources

Esra Mutlu, Sarah H. Warren, Seth M. Ebersviller, Ingeborg M. Kooter, Judith E. Schmid, Janice A. Dye, William P. Linak, M. Ian Gilmour, James J. Jetter, Mark Higuchi, and David M. DeMarini

Table of Contents

Table S1. Parameters measured at high- and low-power test conditions
Table S2. Pollutant-emission factors derived from analyses of organic extracts of $PM_{2.5}$
Table S3. Mutagenicity in *Salmonella* of organic extracts of $PM_{2.5}$ from the three emissions
Table S4. Mutagenic potencies of EOM (rev/µg EOM ± SE)
Table S5. Mutagenic potencies of $PM_{2.5}$ (rev/mg $PM_{2.5}$ ± SE)
Table S6. Comparison of emission factors from replicate experiments expressed as useful energy delivered to the pot (MJ_d)
Table S7. Comparison of emission factors from replicate experiments expressed as fuel energy used (MJ_{th})
Table S8. Comparison of mutagenicity- and pollutant-emission factors between replicate experiments for FDS
Table S9. Pearson correlation coefficients among emission factors expressed per MJ_d

Table S1. Parameters measured at high- and low-power test conditions

			Emission source[b]			
Pollutant[a]	Performance measure	Units	TSF	NDS	FDS	Propane
None	High-power fuel burning rate	g/min	15.3	13.6	14.3	3.1
	High-power time to boil	Min	37	34	22	22
	Low-power fuel burning rate	g/min	9.1	10.8	5.6	1.9
	Low-power test time	Min	45	45	45	45
$PM_{2.5}$	High-power emission rate	mg/h	1926	1266	444	10.8
	Low-power emission rate	mg/h	918	378	228	1.8
CO	High-power emission rate	g/h	34.2	15.6	6.0	1.8
	Low-power emission rate	g/h	39	18	3.6	0.12
THC	High-power emission rate	g/h	4.86	3.0	0.84	0.18
	Low-power emission rate	g/h	2.28	0.72	0.18	0.018
CH_4	High-power emission rate	g/h	1.14	0.54	0.18	0.012
	Low-power emission rate	g/h	0.60	0.18	0.06	0.000
NO_x	High-power emission rate	g/h	0.66	0.72	0.66	0.24
	Low-power emission rate	g/h	0.48	0.48	0.18	0.12
BC	High-power emission rate	mg/h	1368	474	198	0.00
	Low-power emission rate	mg/h	258	108	120	0.00

[a]Emission rates for $PM_{2.5}$ derived from samples collected on filters; rates for other pollutants derived from continuous-emission monitoring. $PM_{2.5}$, particulate material ≤ 2.5 μm in diameter; CO, carbon monoxide; THC, total hydrocarbons; CH_4, methane; NO_x, oxides of nitrogen; BC, black carbon.

[b]TSF, three-stone fire; NDS, natural-draft stove; FDS, forced-draft stove.

Table S2. Pollutant-emission factors derived from analyses of organic extracts of $PM_{2.5}$[a]

Chemical[b]	μg /g particle			μg /MJ_{th}			μg /MJ_d			μg /kg fuel			μg /h		
	TSF	NDS	FDS	TSF	NDS	FDS	TSF	NDS	FDS	TSF	NDS	FDS	TSF	NDS	FDS
Naphthalene	11.7	10.7	9.9	1.2	0.7	0.3	5.2	2.1	0.8	18.0	9.9	4.5	16.3	8.3	2.7
Acenaphthylene	6.9	9.8	6.5	0.7	0.6	0.2	3.1	1.9	0.5	10.6	9.0	2.9	9.6	7.5	1.8
Acenaphthene	<	<	<	0.0	0.0	0.0	0.0	0.0	0.0	0.0	0.0	0.0	0.0	0.0	0.0
Fluorene	4.1	5.4	2.6	0.4	0.3	0.1	1.8	1.1	0.2	6.3	4.9	1.2	5.7	4.1	0.7
Phenanthrene	328.4	170.7	54.1	35.1	10.9	1.5	147.4	34.0	4.2	507.4	157.2	24.5	458.4	131.4	14.8
Anthracene	74.2	44.9	10.7	7.9	2.9	0.3	33.3	8.9	0.8	114.6	41.3	4.8	103.5	34.5	2.9
Fluoranthene	3178.0	1901.9	426.5	340.0	121.7	12.1	1426.9	378.5	33.4	4910.0	1751.6	193.6	4436.4	1464.4	116.4
Pyrene	3919.5	2487.1	479.8	419.4	159.2	13.6	1759.9	494.9	37.6	6055.6	2290.6	217.8	5471.6	1915.0	131.0
Benzo[*a*]anthracene	1747.9	1658.1	517.9	187.0	106.1	14.7	784.8	330.0	40.6	2700.5	1527.1	235.1	2440.0	1276.7	141.4
Chrysene	1589.0	1463.0	609.3	170.0	93.6	17.3	713.5	291.1	47.7	2455.0	1347.4	276.6	2218.2	1126.5	166.3
Benzo[*b*]fluoranthene	1377.1	1267.9	533.1	147.4	81.1	15.1	618.3	252.3	41.7	2127.6	1167.8	242.0	1922.5	976.3	145.5
Benzo[*k*]fluoranthene	1271.2	1365.5	510.3	136.0	87.4	14.5	570.8	271.7	40.0	1964.0	1257.6	231.7	1774.6	1051.4	139.3
Benzo[*a*]pyrene	1959.7	1804.3	571.2	209.7	115.5	16.2	879.9	359.1	44.7	3027.8	1661.8	259.3	2735.8	1389.3	155.9
Indeno[*123-cd*]pyrene	1218.2	1170.4	380.8	130.3	74.9	10.8	547.0	232.9	29.8	1882.2	1077.9	172.9	1700.6	901.2	104.0
Dibenzo[*ah*]-anthracene	58.3	58.5	23.6	6.2	3.7	0.7	26.2	11.6	1.8	90.0	53.9	10.7	81.3	45.1	6.4
Benzo[*ghi*]-perylene	1271.2	1267.9	411.3	136.0	81.1	11.7	570.8	252.3	32.2	1964.0	1167.8	186.7	1774.6	976.3	112.3
**∑ EPA PAHs**	**18015.4**	**14686.1**	**4547.6**	**1927.3**	**939.7**	**129.1**	**8088.9**	**2922.4**	**356.0**	**27833.6**	**13525.8**	**2064.3**	**25149.1**	**11308.0**	**1241.4**
1,4-Naphthoquinone	22.8	15.6	12.2	2.4	1.0	0.3	10.2	3.1	1.0	35.2	14.4	5.5	31.8	12.0	3.3
1-Naphthalene-carboxaldehyde	10.6	7.3	<	1.1	0.5	0.0	4.8	1.5	0.0	16.4	6.7	0.0	14.8	5.6	0.0
9-Fluorenone	141.9	66.3	48.0	15.2	4.2	1.4	63.7	13.2	3.8	219.3	61.1	21.8	198.2	51.1	13.1
9,10-Anthraquinone	321.0	153.1	163.0	34.3	9.8	4.6	144.1	30.5	12.8	495.9	141.0	74.0	448.1	117.9	44.5
1,8-Naphthalic anhydride	520.1	287.7	267.3	55.7	18.4	7.6	233.5	57.3	20.9	803.6	265.0	121.4	726.1	221.5	73.0
9,10-Phenanthrene-quinone	<	<	<	0.0	0.0	0.0	0.0	0.0	0.0	0.0	0.0	0.0	0.0	0.0	0.0
Benzanthrone	937.0	867.1	692.3	100.3	55.5	19.7	420.7	172.5	54.2	1447.6	798.6	314.3	1308.0	667.6	189.0
1-Pyrene-carboxaldehyde	46.6	43.4	38.1	5.0	2.8	0.3	20.9	2.1	3.0	72.0	40.0	17.3	65.1	33.4	10.4
Benz[a]anthracene-7,12-quinone	53.5	42.4	68.5	5.7	2.7	0.2	24.0	1.9	5.4	82.7	39.1	31.1	74.7	32.7	18.7
**∑ Oxy-PAHs**	**2053.5**	**1482.9**	**1289.4**	**219.7**	**94.9**	**34.1**	**921.9**	**282.1**	**101.1**	**3172.7**	**1365.9**	**585.4**	**2866.8**	**1141.8**	**352.0**
**∑ Total PAHs**	**20068.9**	**16169.0**	**5837.0**	**2147.0**	**1034.6**	**163.2**	**9010.8**	**3204.5**	**457.1**	**31006.3**	**14891.7**	**2649.7**	**28015.9**	**12449.8**	**1593.4**
Levoglucosan	264.8	73.1	30.5	28.3	4.7	0.9	118.9	14.6	2.4	409.2	67.4	13.8	369.7	56.3	8.3

[a]TSF, three-stone fire; NDS, natural-draft stove; FDS, forced-draft stove; MJ_{th}, megajoule thermal energy; MJ_d, megajoule energy delivered to the cooking pot.

[b]The first 16 chemicals are the EPA priority PAHs; the next 9 are oxy-PAHs; levoglucosan is a marker of wood smoke

Table S3. Mutagenicity in *Salmonella* of organic extracts of $PM_{2.5}$ from the three emissions

		Rev/plate[a]					
	µg EOM	TSF		NDS		FDS	
Strain	per plate	+S9	-S9	+S9	-S9	+S9	-S9
TA100	0	109[b]	87	109[b]	87	117	90
	1.0	NA	NA	NA	NA	225	NA
	2.5	169	NA	227	NA	373	159[c]
	5	289[b]	121	421[b]	134[c]	510	166
	10	399	163	552	238	765[c,d]	229
	20	NA	NA	NA	NA	NA	330[c]
	25	1105[c]	246	1313[c]	398	NA	NA
	50	1309[c,d]	NA	1565[c,d]	817[c]	NA	NA
TA98	0	58	47[b]	58	47[b]	51	37
	1.0	NA	NA	NA	NA	NA	44[c]
	2.5	NA	NA	NA	NA	125	79
	5	106	62[b]	135	93[b]	192	119
	10	NA	NA	138[c]	NA	262[d]	178[d]
	20	NA	NA	NA	NA	NA	NA
	25	246	143[b]	406	207[b]	NA	NA
	50	416	193[b,d]	666	337[b]	NA	NA
TA104	0	276	276[c]	276	276[c]	306	194[c]
	2.5	NA	NA	NA	NA	446	237[c]
	5	365	284[c]	439[c]	327[c]	565	312[c]
	10	440	332[c]	552	360[c]	647[d]	358[c,d]
	20	NA	NA	NA	NA	705[c,d]	402[c,d]
	25	608	402[c]	806[d]	559[c]	NA	NA
	50	NA	NA	1018[c,d]	NA	NA	NA
YG1041	0	50[c]	56[b]	50[c]	56	54[c]	36
	0.25	NA	NA	NA	NA	55[c]	82
	0.5	NA	NA	NA	NA	78[c]	125
	1	NA	125[c]	69[c]	102	110[c]	215
	2.5	78[c]	162[b]	127[c]	198	NA	NA
	5	144[c]	277	249[c]	345	NA	NA
	10	263[c]	433	NA	513[d]	NA	NA

[a]TSF, three-stone fire; NDS, natural-draft stove; FDS, forced-draft stove; rev, revertants; EOM, extractable organic material; except where noted, data are the average of 2 independent mutagenicity experiments, each at 1 plate per dose. Thus, unless noted otherwise, data are the average of 2 plates/dose. NA, not applicable; these doses were not tested. Positive controls data (average rev/plate, range) are 2-aminoanthracene (+S9): TA100 (719, 446-1046), TA98 (576, 436-702), YG1041 (1483, 1221-1608),

TA104 (647, 608-688); sodium azide (-S9): TA100 (646, 548-848); 2-nitrofluorene (-S9): TA98 (522, 394-664), YG1041 (1383, 1240-1497); and methylglyoxal (-S9): TA104 (424, 382-462).

[b]Data are the average of 3 independent mutagenicity experiments, each at 1 plate per dose; thus, the data are the average of 3 plates.

[c]Data are from a single experiment with 1 plate per dose.

[d]These data were not used in the linear regressions because they were outside of the linear portion of the dose-response curves.

Table S4. Mutagenic potencies of EOM (rev/μg EOM ± SE)[a]

Strain	TSF		NDS		FDS	
	+S9	-S9	+S9	-S9	+S9	-S9
TA100	36.9 ± 1.9	6.3 ± 0.5	45.2 ± 2.9	13.7 ± 0.9	77.8 ± 5.9	12.3 ± 1.2
TA98	7.1 ± 0.5	3.4 ± 0.4	12.1 ± 2.0	5.7 ± 0.4	23.2 ± 2.3	14.4 ± 1.1
TA104	16.2 ± 0.7	5.3 ± 0.62	20.4 ± 3.9	11.4 ± 0.9	51.8 ± 6.6	23.6 ± 3.7
YG1041	22.0 ± 1.9	37.9 ± 2.1	40.9 ± 4.0	49.0 ± 3.6	60.0 ± 9.8	178.9 ± 14.2

[a]TSF, three-stone fire; NDS, natural-draft stove; FDS, forced-draft stove; EOM, extractable organic material; data are slopes of linear regressions calculated from the data in Table S3.

Table S5. Mutagenic potencies of $PM_{2.5}$ (rev/mg $PM_{2.5}$ ± SE)[a]

	TSF		NDS		FDS	
Strain	+S9	-S9	+S9	-S9	+S9	-S9
TA100	12391.7 ± 635.0	2126.9 ± 164.6	8083.6 ± 524.5	2450.5 ± 153.9	2333.1 ± 176.7	369.9 ± 36.9
TA98	2378.9 ± 154.6	1139.0 ± 127.7	2162.3 ± 241.7	1020.3 ± 66.2	695.7 ± 69.3	430.5 ± 33.3
TA104	5439.8 ± 221.76	1777.4 ± 208.3	3646.2 ± 699.9	2040.6 ± 162.9	1554.0 ± 198.6	708.0 ± 111.0
YG1041	7398.7 ± 648.5	12731.0 ± 715.7	7313.9 ± 716.0	8771.0 ± 637.2	1800.0 ± 292.5	5365.8 ± 426.6

[a]TSF, three-stone fire; NDS, natural-draft stove; FDS, forced-draft stove; EOM, extractable organic material. The values were calculated by first multiplying the rev/µg EOM (Table S4) by 1000 to give rev/mg EOM. Then the rev/mg EOM was multiplied by the %EOM for each stove as described in the Methods to give rev/mg $PM_{2.5}$. The %EOM values were 33.6% for TSF, 17.9% for NDS, and 3% for FDS.

Table S6. Comparison of emission factors from replicate experiments expressed as useful energy delivered to the pot (MJ_d)[a]

Stove	Parameter	Units	Value	SD	CV (%)	n	SE
TSF	Fuel-burn rate	g/min	12.96	1.05	8	4	0.5
	CO_2	g/MJ_d	393.4	18.4	5	4	9.2
	$PM_{2.5}$	mg/MJ_d	449.4	50.0	11	4	25.0
	CO	g/MJ_d	11.8	0.8	7	4	0.4
	THC	g/MJ_d	1.13	0.22	19	4	0.11
	CH_4	g/MJ_d	0.31	0.04	13	4	0.02
	BC	mg/MJ_d	254.0	42.5	17	4	21.3
NDS	Fuel-burn rate	g/min	12.39	0.49	4	4	0.2
	CO_2	g/MJ_d	310.8	6.3	2	4	3.2
	$PM_{2.5}$	mg/MJ_d	198.6	138.7	70	4	69.4
	CO	g/MJ_d	4.2	0.5	12	4	0.3
	THC	g/MJ_d	0.47	0.18	38	4	0.09
	CH_4	g/MJ_d	0.09	0.03	33	4	0.02
	BC	mg/MJ_d	68.0	15.6	23	2	11.0
FDS	Fuel-burn rate	g/min	11.89	0.84	7	3	0.5
	CO_2	g/MJ_d	244.9	16.1	7	3	9.3
	$PM_{2.5}$	mg/MJ_d	78.2	30.6	39	3	17.7
	CO	g/MJ_d	1.2	0.1	8	3	0.1
	THC	g/MJ_d	0.11	0.07	64	3	0.04
	CH_4	g/MJ_d	0.03	0.01	33	3	0.01
	BC	mg/MJ_d	42.3	4.5	11	3	2.6
Propane	Fuel-burn rate	g/min	2.61	0.00	0	4	0.00
	CO_2	g/MJ_d	96.1	0.5	< 1	4	0.3
	$PM_{2.5}$	mg/MJ_d	4.2	0.5	12	4	0.3
	CO	g/MJ_d	0.2	0.0	0	4	0.0
	THC	g/MJ_d	0.02	0.02	100	4	0.01
	CH_4	g/MJ_d	0.00	0.00	NA[b]	4	0.00
	BC	mg/MJ_d	0.0	0.0	NA[b]	4	0.0

[a]Pollutant-emission factors calculated as weighted averages as described in Methods. The n represents the number of replicate combustion experiments. MJ_d, megajoule energy delivered to the cooking pot; TSF, three-stone fire; NDS, natural-draft stove; FDS, forced-draft stove; CO_2, carbon dioxide; $PM_{2.5}$, particulate material ≤ 2.5 μm in diameter; THC, total hydrocarbons; CH_4, methane; BC, black carbon.
[b]When the value is 0, the CV cannot be calculated.

Table S7. Comparison of emission factors from replicate experiments expressed as fuel energy used (MJ_{th})[a]

Stove	Pollutant	Units	Value	SD	CV (%)	n	SE
TSF	CO_2	g/MJ_{th}	93.8	1.5	2	4	0.8
	$PM_{2.5}$	mg/MJ_{th}	102.1	11.8	12	4	5.9
	CO	g/MJ_{th}	3.0	0.4	12	4	0.2
	THC	g/MJ_{th}	0.26	0.05	19	4	0.03
	CH_4	g/MJ_{th}	0.07	0.01	14	4	0.01
	BC	mg/MJ_{th}	51.1	6.6	13	4	3.3
NDS	CO_2	g/MJ_{th}	91.3	0.6	1	4	0.3
	$PM_{2.5}$	mg/MJ_{th}	56.9	14.1	25	4	7.1
	CO	g/MJ_{th}	1.3	0.2	12	4	0.1
	THC	g/MJ_{th}	0.13	0.05	38	4	0.03
	CH_4	g/MJ_{th}	0.03	0.01	33	4	0.01
	BC	mg/MJ_{th}	38.3	3.3	9	2	2.3
FDS	CO_2	g/MJ_{th}	90.0	0.7	1	3	0.4
	$PM_{2.5}$	mg/MJ_{th}	28.4	10.7	38	3	6.2
	CO	g/MJ_{th}	0.4	0.0	0	3	0.0
	THC	g/MJ_{th}	0.04	0.03	75	3	0.02
	CH_4	g/MJ_{th}	0.01	0.00	0	3	0.00
	BC	mg/MJ_{th}	15.1	1.3	9	3	0.8
Propane	CO_2	g/MJ_{th}	62.3	0.4	1	4	0.2
	$PM_{2.5}$	mg/MJ_{th}	0.8	0.6	75	4	0.3
	CO	g/MJ_{th}	0.1	0.0	0	4	0.0
	THC	g/MJ_{th}	0.01	0.01	100	4	0.01
	CH_4	g/MJ_{th}	0.00	0.00	NA[b]	4	0.00
	BC	mg/MJ_{th}	0.0	0.0	NA[b]	4	0.0

[a]Pollutant-emission factors calculated as weighted averages as described in Methods. The n represents the number of replicate combustion experiments. MJ_{th} megajoule thermal energy; TSF, three-stone fire; NDS, natural-draft stove; FDS, forced-draft stove; CO_2, carbon dioxide; $PM_{2.5}$, particulate material ≤ 2.5 µm in diameter; THC, total hydrocarbons; CH_4, methane; BC, black carbon.

[b]When the value is 0, the CV cannot be calculated.

Table S8. Comparison of mutagenicity- and pollutant-emission factors between replicate experiments for FDS[a]

Parameter	Experiment 1	Experiment 2	*p*-value
% EOM	2.3	3.0	
mg $PM_{2.5}/MJ_d$	88.3	78.3	0.629[b]
g CO/MJ_d	1.1	1.2	0.449 [b]
g THC/MJ_d	0.06	0.11	0.344 [b]
g CH_4/MJ_d	0.02	0.03	0.494 [b]
g BC/MJ_d	56.3	42.3	0.039 [b]
TA98+S9 rev/µg EOM	25.0	28.2	0.630[c]
TA98+S9 rev x $10^5/MJ_d$	0.5	0.6	
TA100+S9 rev/µg EOM	98.2	77.8	0.305[c]
TA100+S9 rev x $10^5/MJ_d$	2.1	1.8	
TA98-S9 rev/µg EOM	29.5	17.2	0.000[c]
TA98-S9 rev x $10^5/MJ_d$	0.6	0.4	
YG1041-S9 rev/µg EOM	333.0	178.5	0.013[c]
YG1041-S9 rev x $10^5/MJ_d$	6.8	4.2	

[a]FDS, forced-draft stove; EOM, extractable organic material; $PM_{2.5}$, particulate material ≤ 2.5 µm in diameter; MJ_d, megajoule energy delivered to the cooking pot; CO, carbon monoxide; THC, total hydrocarbons; CH_4, methane; BC, black carbon.

[b]All pollutant-emission factors were calculated as weighted averages as described in Methods. Values were compared using a two-tailed Student's t-test with Welch's correction ($\alpha = 0.05$).

[c]We calculated linear regressions for each of two independent experiments consisting of 2 independent mutagenicity measurements per experiment and then compared the slopes of the regression lines using Statgraphics Centurion XVI (Statpoint Technologies, Inc., Warrenton, VA) as follows. The data from the two groups were analyzed in a multiple-regression model that allowed for a separate intercept and slope for each group. A t-test was run within the model to test for a difference between the two slopes. The mutagenicity-emission factors (rev/MJ_d) were calculated as described in the Methods; the formula contains several standard and calculated values, and consequently, there is no measure of precision or variance in the final value. Thus, no *p*-value can be calculated for the mutagenicity-emission factors.

Table S9. Pearson correlation coefficients among emission factors expressed per MJ_d

	Correlations[a]									
Emission Factors	Rev TA100+S9	Total PAHs	EPA PAHs	Oxy-PAHs	Levo-Glucosan	$PM_{2.5}$	CO	THC	CH_4	NO_x
Total PAHs	1.00									
EPA PAHs	1.00	1.00								
Oxy-PAHs	1.00	0.99	0.99							
Levoglucosan	0.99	0.97	0.97	0.99						
$PM_{2.5}$	1.00	1.00	1.00	0.99	0.97					
CO	1.00	1.00	1.00	1.00	0.98	1.00				
THC	1.00	1.00	1.00	1.00	0.98	1.00	1.00			
CH_4	1.00	1.00	1.00	0.99	0.97	1.00	1.00	1.00		
NO_x	0.70	0.75	0.75	0.67	0.58	0.75	0.73	0.73	0.76	
BC	0.99	0.98	0.98	1.00	1.00	0.98	0.98	0.98	0.98	0.59

[a]Correlations were based on MJ_d (megajoule energy delivered to the cooking pot) for TSF (three-stone fire, NDS (natural-draft stove), and FDS (forced-draft stove) from Tables 1, 2, and Table S2. Analyses of CO (carbon monoxide), THC (total hydrocarbons), CH_4 (methane), NO_x (oxides of nitrogen), and BC (black carbon) were derived from samples collected in real time, whereas the $PM_{2.5}$ (particulate material ≤ 2.5 µm in diameter) was collected on filters; the PAHs (polycyclic aromatic hydrocarbons), levoglucosan, and mutagenicity were determined from DCM (dichloromethane) extracts of the collected $PM_{2.5}$.
